# Effect of surgical traumas on microcirculation

**DOI:** 10.4103/0970-0358.59271

**Published:** 2009

**Authors:** Visweswar Bhattacharya, Biswajit Mishra, Binayak Mishra, Umesh Kumar, Siddhartha Bhattacharya

**Affiliations:** Department of Plastic Surgery, Institute of Medical Sciences, Banaras Hindu University, Varanasi, Uttar Pradesh-221005, India

**Keywords:** Microcirculation, surgical trauma, cautery trauma, clamp trauma, forceps trauma

## Abstract

**Background:**

Adequate microcirculation in different tissues maintains the physiological function and heals surgical wounds. In any surgical procedure, the commonly used instruments are cautery, tissue forceps, and clamps. The fact that their inappropriate use produces an adverse effect on microcirculation is often not realized. By this study, we could demonstrate live, the effect of these surgical traumas.

**Methods:**

The study was conducted on the inferiorly based fasciocutaneous flap with a fascial extension in patients with a distal leg defect. The extended fascial flap was mounted on a glass slide and observed for live microcirculation under ×160 magnification. Three methods were used: (a) cautery in low power, (b) microcrushing forceps to crush the vessels, and (c) noncrushing clamps at the base of the fascial flap.

**Results:**

It was observed that the vessels are well protected within the deep fascia. Once the fascia was pierced the current damaged the vessel wall. As the wattage was increased, it caused charring of the tissue and multiple vessels ultimately leading to cessation of blood flow. Once the vessel wall was crushed by forceps, blood extravasated in a variable intensity depending upon the size of the vessel. Clamping led to gradual slowing of blood flow with microclot formation. In certain vessels, there was discontinuity in the blood column and ultimately the blood flow stopped.

**Conclusion:**

This study showed live demonstration of the effect of surgical traumas on microcirculation. It should guide the surgeons to select the use of appropriate instruments which will cause minimal damage to vascularity and thereby lead to a better surgical outcome.

## INTRODUCTION

Adequate microcirculation is essential for normal physiological function of any organ and success of surgical wound healing. Its disruption beyond a certain limit may lead to partial or complete functional derangement and poor healing. During most surgical procedures, surgeons commonly use three manoeuvers which may adversely affect microcirculation, namely, (a) electric cautery to control bleeding in the field of surgery, (b) forceps, etc., to hold tissue, and (c) different types of clamps to occlude blood flow. These manoeuvers are essential but should be used judiciously applying appropriate instruments keeping in mind that “if you respect the tissues, tissues will respect you”.

Live microcirculation cannot be demonstrated in most of the tissues as they are thick. The tissue has to be thin and transparent to be observed under the microscope. We have already demonstrated and standardized the visualization of live microcirculation in the deep fascia covering the muscles.[1] Therefore, as an extension of this study, we made further observations.

## MATERIAL AND METHODS

On 10 patients, retrograde fasciocutaneous flaps with a fascial extension were dissected in the lower limb for distal leg and foot defects. These flaps were based on the lower perforators of the posterior tibial or peroneal vessels. Beyond the proposed distal margin of the fasciocutaneous flap, a lazy S incision was made from the center of the flap on the proximal calf. The skin and subcutaneous tissue were undermined on either side under loupe magnification preserving the suprafascial vascular plexus. Thereafter, three incisions were made distally in continuity with the fasciocutaneous flap in the deep fascia. The dissection was done through the epimysium and a fascial flap extension of the fasciocutaneous flap was raised. This fascial extension was mounted on a glass slide under natural tension using corner stitches. It was observed under 10× lens having ×160 magnification (Olympus microscope). Live microcirculation was witnessed. Then we intended to visualize the effect of commonly used surgical trauma such as:

- Diathermy
- Crushing of vessels by forceps
- Clamping the pedicle of the fascial flap.

Formal approval was taken from the ethical committee of the institute for this study and written consent from the patients was obtained.

### Diathermy

**Figure 1:**
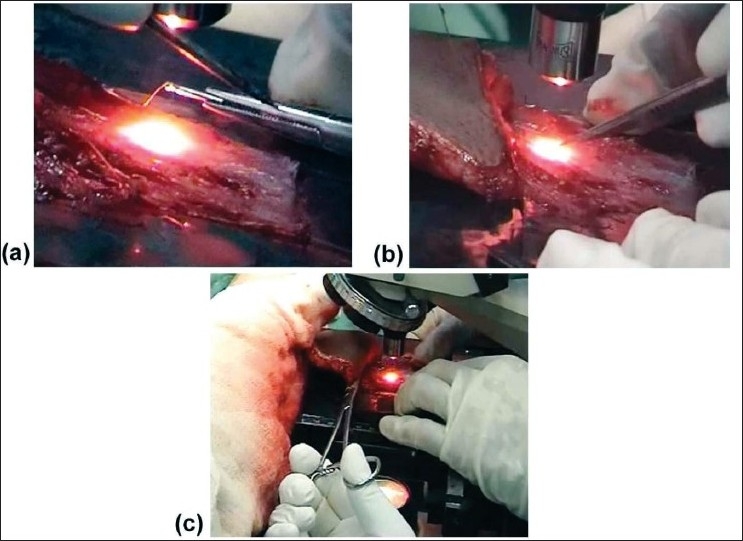
(a) Diathermy probe touching the needle holder with the microneedle in contact with the vessel. (b) The two prongs of the microforceps under ×160 magnification crushing the vessel. (c) The two clamps are in place at the pedicle of the fascial extension flap

A microneedle was held on a needle holder and brought to the fascial flap under the field of vision of the microscope. The vessel with active microcirculation was focused. In an attempt to damage the vessel, we touched it with this needle. The diathermy probe was then touched on the needle holder to transmit the current through the needle to the vessel [Figure 1a]. The diathermy was adjusted to the lowest power (25 – 100 watts).

### Crushing

Microforceps were used to crush vessels of different calibers under vision. The two prongs of the forceps was approximated on either side of the vessel and pressure was applied [Figure 1b].

### Clamping:

While monitoring the circulation, two non-crushing clamps were applied on the pedicle, at half width from either side, at the junction of the proximal fasciocutaneous flap and the distal fascial extension [Figure 1c]. This occluded the flow of blood distal to the clamps in the fascial flap.

## RESULTS

### Effect of diathermy

**Figure 2:**
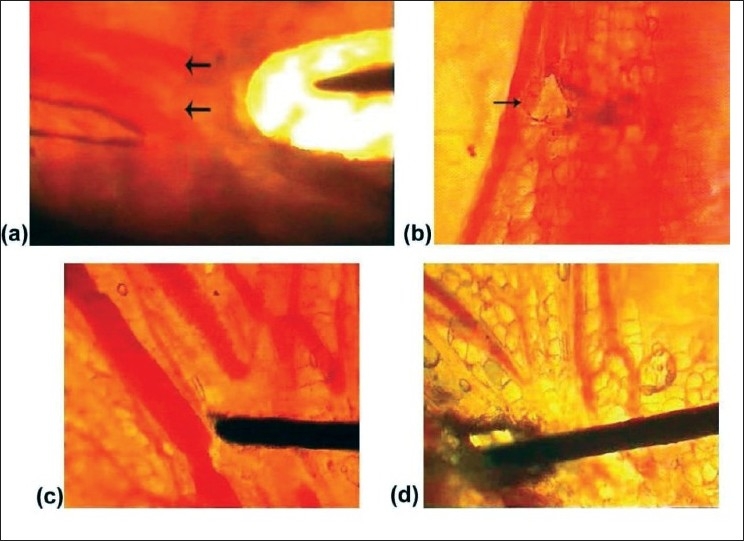
(a) Due to diathermy, there is a hole in the deep fascia along with the damaged blood vessels. (b) Full thickness rent on the wall of a blood vessel. (c) Multiple damaged vessels. (d) Charring of the tissue including vessels.
- Under the effect of current, there was damage of the vessel wall in the form of a rent, suggestive of full thickness damage, and the blood flow stopped [Figure 2b and Video 1.]
- There were multiple vascular damages in a given field [Figure 2c].
- When the power was increased, charring of tissue including vessels [Figure 2d] was observed.
- Flow in the adjacent undamaged vessels continued [Video 2].

The following interesting observations were made:

- The vessels were well protected within the fascia.
- Once the fascia got burnt, enough to form a rent, then only the vessels within them got damaged and the blood flow stopped. The discontinuity of the vessels proximal and distal to the burnt fascia could be seen [Figure 2a].

### Effect of crushing

**Figure 3:**
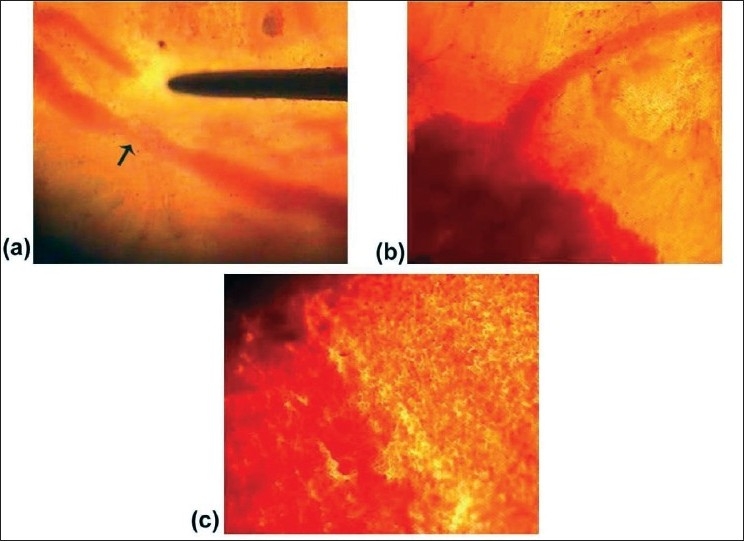
Narrowing of the vessel lumen due to the pressure by forceps. (b) Spilling out of blood from a smaller vessel. (c) Spilling out of blood from a larger vessel.
- Once the fibre of the fascia was pierced and the wall of the vessels got crushed, blood spilled out of the intravascular space to the extravascular space. The intensity varied depending upon the dimension of the vessel. Larger the vessel, more the extravasation of blood [Figure 3b, c and Video 3].

The following effects were seen:

- As pressure was applied by the forceps in an attempt to damage the vessels, the fascia resisted it. The luminal size of vessels got steadily narrow by the external pressure but vessels per se were not damaged and blood flow continued uninterrupted [Figure 3a].

### Effect of clamping

**Figure 4:**
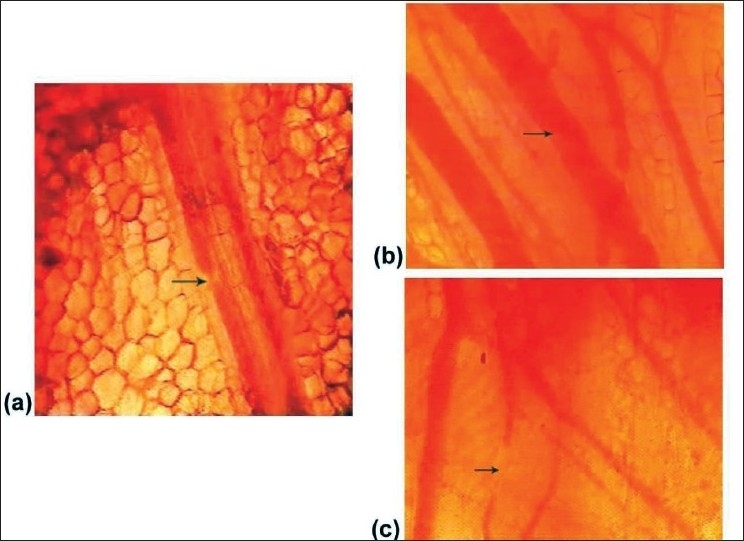
(a) Gradual reduction in the flow of blood. (b) Intravascular microclots. (c) Discontinuity in the blood column.
- Prior to total cessation of the flow, there was rouleaux formation of red cells in the form of microclots which moved sluggishly in a jerky motion [Figure 4b and Video 4].
- On a few occasions, there was discontinuity in the blood column within the lumen of the vessel [Figure 4c].

As soon as the clamps were applied,

- The rate of the flow of blood gradually started reducing until it totally stopped in all the vessels in the field [Figure 4a].

## DISCUSSION

There is scanty information in the literature regarding the live demonstration of surgical traumas on microcirculation in any tissue. In this study, we have used the deep fascia as a model for all types of tissues of our body. It was demonstrated that the deep fascia has rich vascularity, supplied by the underlying vessels, in the form of supra- and subfascial vascular plexuses.[2,3] We have also successfully demonstrated and standardized the live microcirculation in the deep fascia. This tissue was chosen because it is one of the few thin transparent structures which allow the observation of vessels under the microscope. The purpose of this article is not intimidate surgeons but to demonstrate live and reemphasize what changes occur in the vessels due to the commonly used manoeuvers in surgical practice. We have chosen three surgical appliances, namely electric cautery, crushing forceps, and temporary clamping. This study suggests that these methods should be used judiciously using appropriate instrument causing minimum damage to the vascularity. This certainly has a positive implication on wound healing and thereby leads to success of surgery. Quite often more than necessary tissue damage is caused by combination of these appliances which compromises the healing process and results in a poorer quality of scar. The vascular network under observation consisted of arterioles, capillaries, and venules. This is the level where gaseous exchange occurs in the tissues to maintain their physiological functions. Therefore, this study helps in understanding the rationality of appropriate surgical techniques causing minimal damage to vascularity. The effect of diathermy at a high power can be seen routinely by the naked eye as charring of tissue and stoppage of bleeding. One may not realize its effect on the adjacent tissue and the proportionate spectrum of vascular damage.[4] This study helped to identify the intensity of vascular damage at the arteriolar and capillary level. Depending upon the magnitude of surgical trauma, one can witness from minimal damage to the vessel wall to destruction of multiple vessels resulting in cessation of blood flow. The protective deep fascia resists the external pressure as well as the heat. A similar structure protects the vessels all over the body. It is only once the fascial barrier is breached, that vessels may be damaged. Thereafter, the different layers of the vessel wall are damaged by the current and forceps. All these models of trauma ultimately cause cessation of the blood flow.[5] Many vessels may be damaged simultaneously in a given field. Once the vessel is affected, blood coagulates within it. An increase in the wattage of current leads to more devitalization of the tissue. In contrast, if the low wattage diathermy is used only to cauterize selective vessels, maintaining the flow in the adjacent vessels, then adequate perfusion is maintained through the vascular network. That is why one prefers to ligate the bleeding vessel or to apply microclamps on a dissected flap rather than cauterizing them. Cauterization may jeopardize the vascularity in a segment of the flap. Even on the recipient bed of a skin graft, one must exercise judicious caution while using the cautery; or else there may be a patchy loss of graft.[6]

Whenever tissue, including vessels, is crushed by forceps or any other such instrument, blood spills out into the extravascular tissue. This underlines the importance of fine forceps to delicately handle tissues. Since crude forceps cause more damage to tissue including vessels, this utimately compromises wound healing. By applying non-crushing clamps we could see the gradual slowing of the blood flow till it stopped completely. Therefore, according to the need of the procedure, one should consider the use of clamps. All the above observations can usually be demonstrated in animal experimental models. Following such studies it is speculated that in human tissues similar effect will be seen. In this study, we have demonstrated, realtime, the effect of various surgical appliances causing surgical trauma on microcirculation.

## See video on www.ijps.org

Video 1

Video 2

Video 3

Video 4
